# Live Cell Light Sheet Imaging with Low- and High-Spatial-Coherence Detection Approaches Reveals Spatiotemporal Aspects of Neuronal Signaling

**DOI:** 10.3390/jimaging9060121

**Published:** 2023-06-16

**Authors:** Mariana Potcoava, Donatella Contini, Zachary Zurawski, Spencer Huynh, Christopher Mann, Jonathan Art, Simon Alford

**Affiliations:** 1 Department of Anatomy and Cell Biology, University of Illinois at Chicago, 808 South Wood Street, Rm 578 MC 512, Chicago, IL 60612, USA; contini@uic.edu (D.C.); zurawski@uic.edu (Z.Z.); spencerh3438@gmail.com (S.H.); jart@uic.edu (J.A.); sta@uic.edu (S.A.); 2 Department of Applied Physics and Materials Science, Northern Arizona University, Flagstaff, AZ 86011, USA; christopher.mann@nau.edu; 3 Center for Materials Interfaces in Research and Development, Northern Arizona University, Flagstaff, AZ 86011, USA

**Keywords:** fluorescence microscopy, lattice light sheet holography, incoherent lattice light sheet, electrophysiology and simultaneous imaging, in situ recording, synaptic function, tomographic imaging

## Abstract

Light sheet microscopy in live cells requires minimal excitation intensity and resolves three-dimensional (3D) information rapidly. Lattice light sheet microscopy (LLSM) works similarly but uses a lattice configuration of Bessel beams to generate a flatter, diffraction-limited z-axis sheet suitable for investigating subcellular compartments, with better tissue penetration. We developed a LLSM method for investigating cellular properties of tissue in situ. Neural structures provide an important target. Neurons are complex 3D structures, and signaling between cells and subcellular structures requires high resolution imaging. We developed an LLSM configuration based on the Janelia Research Campus design or in situ recording that allows simultaneous electrophysiological recording. We give examples of using LLSM to assess synaptic function in situ. In presynapses, evoked Ca^2+^ entry causes vesicle fusion and neurotransmitter release. We demonstrate the use of LLSM to measure stimulus-evoked localized presynaptic Ca^2+^ entry and track synaptic vesicle recycling. We also demonstrate the resolution of postsynaptic Ca^2+^ signaling in single synapses. A challenge in 3D imaging is the need to move the emission objective to maintain focus. We have developed an incoherent holographic lattice light-sheet (IHLLS) technique to replace the LLS tube lens with a dual diffractive lens to obtain 3D images of spatially incoherent light diffracted from an object as incoherent holograms. The 3D structure is reproduced within the scanned volume without moving the emission objective. This eliminates mechanical artifacts and improves temporal resolution. We focus on LLS and IHLLS applications and data obtained in neuroscience and emphasize increases in temporal and spatial resolution using these approaches.

## 1. Introduction

Lattice light sheets (LLS) were proposed in 2014 [1] as a method of recording live-cell long-term volumetric images with subcellular resolution. LLSs are a form of light sheet fluorescence microscopy (LSFM) systems. LLSs were developed as an imaging technique with good optical-sectioning to record faster and scan larger sample volumes at low radiation over longer time frames [2]. In LLSs, as in other LSFM systems, the emitted fluorescence light is collected by the detection objective in a perpendicular direction from the excitation light and without the need for a confocal aperture as it is done in laser scanning confocal microscopy [3]. This characteristic prevents the excitation light from passing through the entire sample; instead, the sample is illuminated from the side with a thin light sheet (LS) beam. The LLS method was inspired by the usage of two-dimensional (2D) optical lattices at various depth positions within the sample volumes [4,5] and has inspired many research ideas since then. The lattice light sheet is formed by superposing a linearly polarized sheet of light with a binary phase map of Bessel beams on a binary spatial light modulator (SLM), which is conjugated to the image plane of the excitation objective. Before reaching the sample plane, the beam passes a Fraunhofer lens and is further projected onto a transparent optical annulus to eliminate unwanted diffraction orders and lengthen the light sheet. In dithered mode, a 2D lattice pattern is oscillated in the x-axis using a galvanometer (x-galvo) to create a uniform sheet, while another galvanometer mirror moves together with the detection objective, in the z-axis (z-galvo), to scan the sample volume. The axial resolution for the dithering mode is limited to the thickness of the light sheet, which is about 400 nm.

We have implemented several aspects of LLSM to allow cellular imaging in intact tissue using low- and high-spatial coherence detection approaches to reveal the spatiotemporal aspects of neuronal signaling. Some of these aspects are described in the following sections of this article. For example, one aspect included the capability of carrying out simultaneous electrophysiological and LLSM imaging [6,7]. Fluorescence microscopy provides a cornerstone of modern biomedical sciences. This approach provides both functional and structural information about cells, tissues, and organs. The complexity of signaling and connectivity in the nervous system make this approach even more important in neuroscience. Since the development of confocal microscopy, fluorescence imaging has also enabled structure and function to be interpreted in 3D space. The basic properties of pinhole/laser scanning confocal imaging limit the speed of data acquisition, although numerous improvements have been implemented, including line scanning [8], spinning disk [9], and, recently, simultaneous multiplane slit scanning [10]. Light sheet microscopy enables a similar 3D view of tissue samples [11] but with two substantial advantages. Firstly, unlike conventional optical arrangements, only the imaged area is exposed to substantial excitation light intensity, and secondly, entire image planes can be captured using standard camera technology, allowing for much higher temporal resolutions than point scanning systems. The LLS approach provides a much thinner excitation sheet using Bessel beams [1]. The sheet also penetrates uncleared tissue better than conventional light sheet techniques. We have sought to use LLSM to image dynamic physiological changes measured with fluorescent probes in tissue in situ [6,7]. This in situ tissue target is important in understanding function and disease in development and aging. While the approach has principally been used to investigate cells in culture, these do not recapitulate developmental stages accurately and cannot be used to investigate the changes in cells during aging. However, tissue can be extracted from animals at any stage of development or age. In the brain, tissue may be kept alive and relatively intact over periods of hours [12] to days [13]. We have used tissue from the central nervous systems of fish [6] and rodents [7] to demonstrate imaging capabilities of LLSM at different temporal resolutions.

A more recent approach of LLSM is the new detection path that uses the incoherent holography idea [14,15], called incoherent holographic lattice light sheet (IHLLS) [15,16,17,18,19], to scan the sample volume without moving the detection objective. This is made possible by projecting a couple of diffractive lenses at various z-galvo positions, using a phase SLM, to encode the depth position of the sample being imaged. Through our work, we showed that IHLLS 2L allows imaging with better transverse and axial resolutions than using the conventional LLS detection system. We have also demonstrated that IHLLS gives access to the 3D amplitude and phase information that can be extracted from fluorescent neuronal cells.

**Figure 1:**
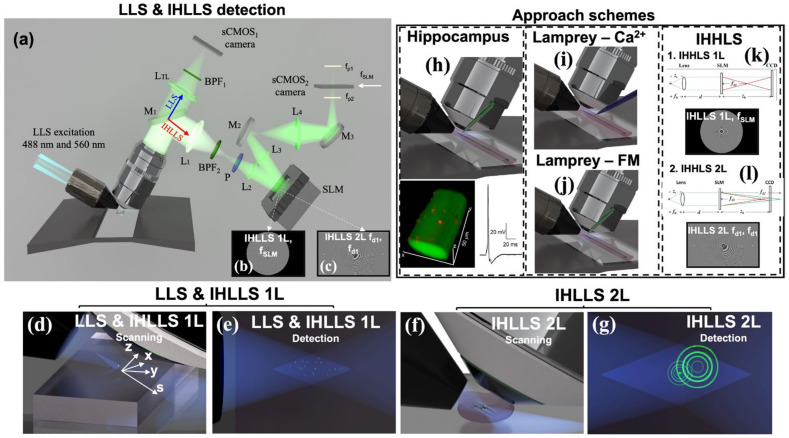
The LLS and IHLLS detection systems. (**a**) Schematics of both detection systems, LLS (blue arrow) and IHLLS (red arrow). Both systems share the water-immersed microscope objective MO (Nikon 25X, NA 1.1, WD 2 mm, Nikon Corporation, Tokyo, Japan). The LLS system consists of a glass-based tube lens, L_TL_ = 500 mm; a Semrock FF01-446/523/600/677-25 bandpass filter, BPF_1_ (IDEX Health & Science, West Henrietta, NY, USA); and a Hamamatsu *ORCA*-Flash 4.0 v3 sCMOS. The diffraction mask in the LLS excitation path was centered for all experiments on an anulus with higher NA, NAout = 0.55 and NAin = 0.485; therefore, the beams used for excitation were more Bessel-like beams. The IHLLS system is equipped with a phase spatial light modulator SLM (1920 × 1152, 9.2 µm pixel size, Meadowlark Inc., Longmont, CO, USA); lenses L_1_ = L_4_ with focal lengths of 175 mm and L_2_ = L_3_ with focal lengths of 100 mm; mirrors M_1_ (sliding mirror), M_2_, M_3_; polarizer P; band pass filters BPF_2_ centered at 575 nm (Chroma Technology Corp., Bellows Falls, VA, USA., 23 nm bandpass width) for the excitation wavelength λ = 488 nm. The camera in the incoherent arm is another Hamamatsu *ORCA*-Flash 4.0 v3 sCMOS: (**b**) one diffractive lens of focal length f_SLM_ = 415 mm at the phase shift θ_1_ = 0 and (**c**) two diffractive lenses with focal lengths f_d1_ = 228 mm and f_d2_ = 2444 mm at the phase shift θ_1_ = 0. Panels (**d**,**e**) show the scanning and detection geometries for the LLS and IHLLS 1L techniques. The vectors represent the x, y, z, and s planes of the Bessel beams that were focused by an excitation objective lens (not showing) to form a lattice light sheet at the sample plan. z and x are moved by the z- and x-galvos. Panels (**f**,**g**) show the scanning and detection geometries for the IHLLS 2L technique. While the z-galvo and z-piezo are moved along the *z* axis to acquire stacks in LLS (**d**,**e**), in IHLLS 2L, only the z-galvo is moved at various z positions. The system performances of all three techniques are shown in Figure 2. The approach schemes for the three experiments are illustrated in panels (**h**–**l**). The first three approaches demonstrate the ability to combine LLSM imaging within situ electrophysiology (**h**–**j**). (**h**) Hippocampal neuron imaging demonstrates the ability to resolve very sparse synaptic inputs to the dendrites of pyramidal cells in intact slices in situ. (**i**) Axons in intact lamprey spinal cord were imaged at high speed (330 frames/second) using Ca^2+^ sensitive dye to demonstrate both the spatiotemporal resolution. (**j**) Lamprey-FM demonstrates the ability to investigate stimulus-evoked lipid vesicle fusion and intracellular transport. We then demonstrated holographic approaches to image in situ lamprey presynaptic structures (**k**,**l**). (**k**) IHLLS 1L, used for settings and calibration, and (**l**) IHLLS 2L, used for holographic imaging. (**a**–**g**) adapted from [16].

We acquired the data for this work using a customized LLS system with the two detection arms mentioned above. Figure 1a shows the conventional LLS detection arm with a glass-based tube lens (blue dotted line pathway) and the incoherent detection arm (red dotted line pathway) in which the glass-based tube lens was replaced by a phase spatial light modulator (SLM). 

The light propagates through either pathway, depending on the orientation of the sliding mirror. Twin lenses are necessary before and after the phase SLM to fit the size of the SLM and to match the transverse magnification of 62.5 in both systems. Both detection systems use the excitation technology of the LLS system. IHLLS uses two combinations of diffractive elements displayed on the SLM. The first system, IHLLS 1L, which is the incoherent version of the original LLS, uses one diffractive lens and a constant phase mask with shared pixels. We use this technique for calibration and alignment purposes. The second technique, IHLLS 2L, uses two diffractive lenses of different focal lengths with randomly selected pixels. This configuration gives the option to choose only one polarizer in the system to create the interference between the beams, which implies that lower doses of light could be used to image biological samples. The IHLLS 2L detection arm was built with the goal of acquiring a holographic sample volume by keeping the detection objective fixed while moving the z-galvanometric mirror to certain positions in the sample volume. This indicates that the same synaptic structures whose dynamic properties are investigated with LLSM can be resolved with holography. We are now developing approaches to enable dynamic changes to be rapidly imaged using holography.

This manuscript consists of novel applications of LLS and IHLLS for the real-time live imaging of intact tissue. We demonstrate how dynamic changes in membrane potential can be resolved in milliseconds, Ca^2+^ signals and neurotransmitter secretions can be resolved in milliseconds to tens of milliseconds, and how intracellular lipid compartments can be resolved in seconds and tracked in 3D. Furthermore, using images of neuronal structure, we demonstrate the development of holographic images in light sheet microscopy and discuss how this can improve the spatiotemporal resolution of fluorescent 3D structures.

## 2. Materials and Methods

All experiments were performed in accordance with institutional guidelines. All work on animals was performed according to institutionalization guidelines in an AALAC-approved facility at UIC. The work was approved by the institutional IACUC review committee under protocol number ACC 20-119 on January 2022.

### 2.1. Performance Metrics of LLS, IHLLS 1L, and IHLLS 2L

**Figure 2:**
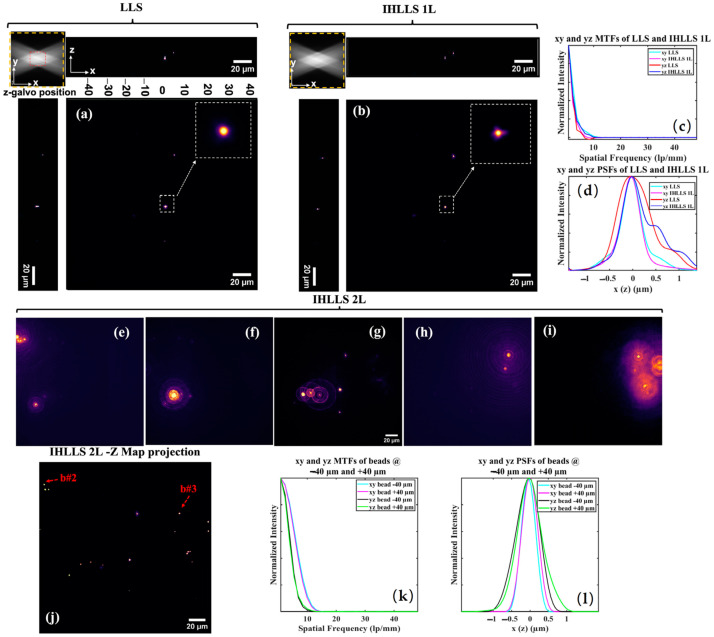
Tomographic imaging of 0.5 µm, FOV 208 µm^2^, in a conventional LLS (**a**) and incoherent LLS with only one diffractive lens (IHLLS 1L) of focal length 400 nm (**b**), without deconvolution. On the sides and above are shown the max projections through the volume (400 z-galvo steps). The Bessel beams are displayed in the upper left corner of each xy-projection to show the orientation of the beams (FOV 208 µm^2^). The area enclosed inside the colored dashed rectangles are as follows: red—the scanning area for the original LLS (52 µm^2^); yellow—the actual scanning area for the LLS, IHLLS 1L, and IHLLS 2L. The bead in the white dashed rectangle that is in the middle of the lattice sheet is considered when calculating the resolution for the two instruments. The 1D xy and yz sections of the MTFs (**c**), the 1D xy and yz of the PSFs (**d**). The FWHM of the curves are cyan, 0.530 µm; magenta, 0.495 µm; red, 0.8341 µm; and blue, 0.9004 µm. IHLLS 2L tomographic beads imaging, (**e**–**i**) holograms at −40 μm (**e**), −30 μm (**f**), 0 μm (**g**), +30 μm (**h**), +40 μm (**i**), and the z-max projection of all of the best z-reconstructed planes (**j**). The max projection of the reconstructed volume of the 500 nm beads sample contains the beads localized at z-galvo levels ±40 μm, ±30 μm, and 0 μm. The 1D xy and yz sections of the MTFs of beads #2 and 3 are shown in (**k**) and the 1D xy and yz of the PSFs of the same beads are shown in (**l**). The FWHM of the curves in (**l**) are cyan, 0.4534 µm; magenta, 0.5118 µm; black, 0.7663 µm; and green, 0.7946 µm.

To fully characterize the performances of the three systems, LLS, IHLLS 1L, and IHLLS 2L, we analyzed the modulation transfer function (MTF) and point spread function (PSF) of fluorescent latex beads (500 nm, λ_exc_ = 488 nm, Molecular Probes, Waltham, MA, USA), Figure 2. We prepared a bead solution (2% solids) diluted with distilled water (1:4000) and centrifuged in a desktop centrifuge for 1 min. A thin layer of bead solution was applied on clean coverslips and left to dry. After drying, the cover slip was mounted on the sample holder under distilled water.

Volumetric imaging of beads was performed using the conventional LLS, Figure 2a, and its incoherent alternative IHLLS 1L, Figure 2b. The scanning area in both systems is localized in the middle of the camera FOV (red dashed rectangle in the upper left corner of Figure 2a), and the Bessel beams cannot excite beads outside that area. The IHLLS 1L and LLS have similar performance metrics, Figure 1c,d, when analyzing these functions on the bead localized in the middle of the FOV. However, LLS performs better than IHLLS 1L in the axial direction due to the blurring effect of one of the lenses that is focused to infinity, Figure 1k.

The IHLLS 2L holograms (phase shift θ = 0) for five z-galvo levels are displayed in Figure 2e–i. The z-galvo levels are ±40 μm, ±30, and 0 μm. We performed scanning at three other *z-galvo* levels, ±10 μm, and ±20 μm, but the results are not shown in Figure 2. The complex hologram is propagated and reconstructed at the best focal plane using a custom diffraction angular spectrum method (ASM) routine programmed in MATLAB R2021b (MathWorks, Inc., Natick, MA, USA). The max projection of all z-planes where the beads were found are displayed in Figure 2j. They show the complex holograms propagated to the best focal plane. We also determined the performance metrics’ distribution, MTF Figure 2k and PSF Figure 2l, of the two beads, bead #2 and bead #3 (Figure 2j), localized at *z-galvo* −40 μm and +40 μm respectively. We notice that IHLLS 2L has better performance metrics than the other two techniques, LLS and IHLLS 2L, due to the better localization of the sample points. We chose the two beads in the image, localized at two extreme edges of the z-galvo range, to check the stability of our detection system, since the beads in the middle of the FOV are always localized at the focal position of the detection objective.

### 2.2. Mouse Hippocampal Slice Experiment

Hippocampal slices (300 µm) were prepared from 21 to 22 d old male and female Sprague Dawley rats anesthetized with isoflurane and decapitated. Hippocampi were isolated under semi-frozen Krebs Henseleit solution (in mm), as follows: 124 NaCl, 26 NaHCO_3_, 1.25 NaH_2_PO_4_, 3 KCl, 2 CaCl_2_, 1 MgCl_2_, and 10 D-glucose; bubbled with 95% O_2_–5% CO_2_; and sliced using a Vibratome (Leica VT1200, Leica Microsystems, Deerfield, IL, USA). All recordings were performed in a constant flow recording chamber in which the slices were held down with a harp. The recording chamber was superfused at ∼2 mL/min and maintained at 28 °C. Neurons were recorded from under whole cell conditions with electrodes containing (in mm) cesium potassium methane sulfonate, 146; MgCl_2_, 2; EGTA, 5; HEPES, 9.1; and pH adjusted to 7.2 with KOH. The electrodes also contained 1 mM QX314 to prevent action potentials, Alexa 647 to label dendritic structure, and 200 μM Oregon Green BAPTA1 to record Ca^2+^ transients. After labeling with dye, the slices were transferred to a perfused recording chamber under the LLSM objective lenses (Figure 1h). The LLSM was designed to allow simultaneous electrophysiology but based on a Janelia Research design [1]. For all experiments, a twisted pair Nichrome stimulating electrode was positioned over the axons of CA3 neurons. Stimulation (200 µs, for 20 shocks at 100 Hz) evoked fluorescence transients within the labeled dendrites. We imaged transients using LLSM at frame rates of up to 80 Hz in single planes. Transients were imaged in sequential LLSM frames at the same z position.

### 2.3. Lamprey Axons Recording

Spinal cords were dissected from ammocoete lampreys (Petromyzon marinus) of either sex, anesthetized with tricaine methanesulfonate (MS-222; 100 mg/L, Sigma-Aldrich, Inc. St. Louis, MO, USA) in ice-cold (4 °C) Ringer’s solution: 130 mm NaCl, 2.1 mm KCl, 2.6 mm CaCl_2_, 1.8 mm MgCl_2_, 4 mm glucose, and 5 mm HEPES, pH 7.6, 270 mOsm. Spinal cords were pinned ventral side up in a cooled (10 °C) perfused recording chamber under the LLSM objectives (Figure 1i).

*Imaging Ca^2+^ transients:* Spinal reticulospinal axons were prelabeled by pressure injecting the Ca^2+^-sensitive dye Fluo5F (5 mM) via a sharp micropipette. A stimulating electrode was positioned over the ventromedial tracts to evoke APs. Stimuli were repeated at 1 min intervals or greater. This evoked Ca^2+^ transients at AZs [20,21]. Transients were imaged in sequential LLSM frames at the same z position.

*Imaging Synaptic Vesicle Recycling using FM1-43;* FM1-43 (5 μm) was applied in the superfusate. Two thousand stimuli were applied to a microelectrode-recorded axon during the dye application (Figure 1j), while the presence of the dye in the tissue surrounding the axons was confirmed by imaging. Dye application was subsequently terminated. During this staining protocol, postsynaptic activity was blocked with glutamate receptor antagonists 6-cyano-7-nitroquinoxaline-2,3-dione (CNQX) and D-amino-phosphono-pentonoate (AP5) (5 and 100 μm, respectively). Excess dye was removed with Advasep 7 [22] to reveal areas of stimuli-dependent staining.

### 2.4. System Control

The entire system was controlled by the original LLS software based on LabView platform (National Instruments Corp., Austin, TX, USA) with the diffractive SLM (Meadowlark Inc., Longmont, CO, USA) synchronized with the ORCA camera mounted in the IHLLS pathway. Subsequent image processing and analysis of the raw data were performed using FIJI/ImageJ tools. In the case of IHLLS, the complex hologram is propagated and reconstructed at the best focal plane using a custom diffraction method routine in MATLAB (MathWorks, Inc.). The laser intensity and detector exposure time in IHLLS 2L were two times higher when compared to the LLS or IHLLS 1L.

## 3. Results

We have demonstrated approaches that highlight the sensitivity, spatiotemporal resolution, and low rates of photodamage while imaging intact tissue. These approaches combine high-spatiotemporal-resolution imaging, low photodamage, and simultaneous electrophysiology. The addition of holographic imaging will allow for low (IHLLS 1L) and high (IHLLS 2L) spatial coherence in intact tissues.

### 3.1. LLS Imaging of AMPA Receptors

**Figure 3:**
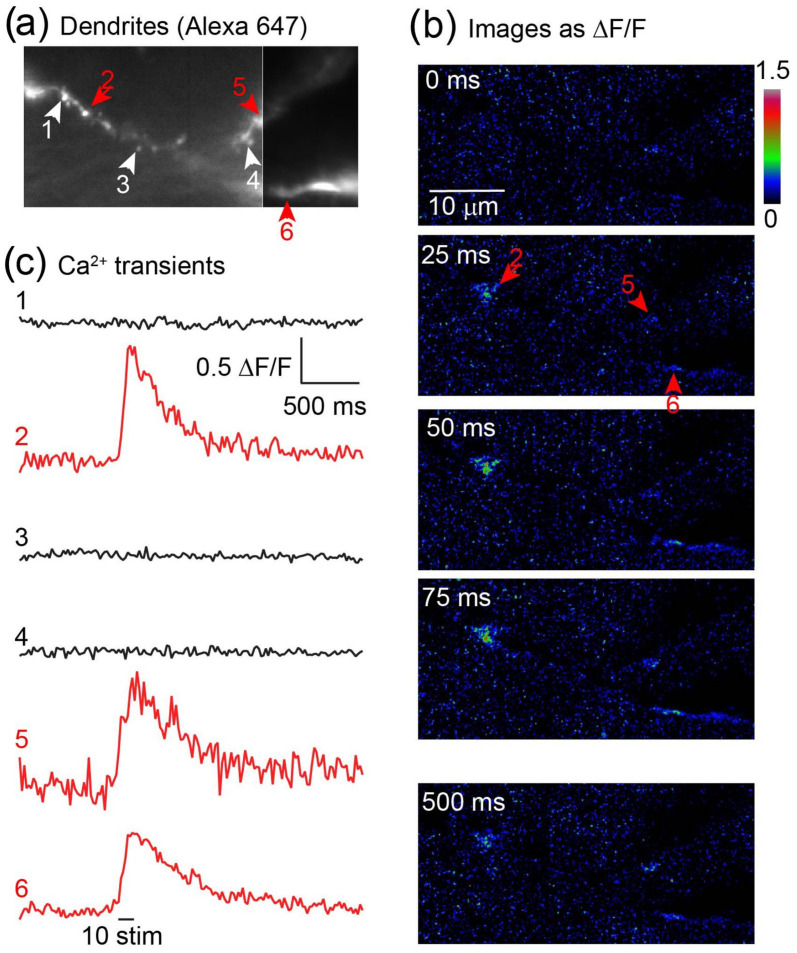
Resolution of synaptic Ca^2+^ responses via Ca^2+^-permeable AMPA receptors in hippocampal CA1 neurons. (**a**) CA1 hippocampal pyramidal neurons in mouse hippocampal slices in situ were whole cell patch clamped with electrodes containing Alexa 647 hydrazide (200 mM), the Ca^2+^-sensitive dye Oregon Green BAPTA1, and the membrane-impermeant Na^+^ channel blocker QX314 to prevent dendritic spiking. Dendrites and dendritic spines were resolved in 3D. (**b**) The tissue was superfused with the NMDA receptor antagonist D-AP5 (100 mM) and imaged in single LLSM planes at 25 ms frame rates. During recording, a train of 10 stimuli applied via a bipolar stimulating electrode over the Schaffer collateral commissural pathway evoked Ca^2+^ transients at discrete sites in the dendritic field, indicated by red arrow heads. (**c**) Measurements were made of the signals both at sites of activity and between. The discrete nature of the signals and the lack of signal between these sites indicates synaptic Ca^2+^ entry. Note that Ca^2+^ entry through Ca^2+^ permeable AMPA receptors has proven hard to resolve in tissue in situ because of the low incidence and Ca^2+^ permeability.

The LLSM’s high spatial and temporal resolution allows the imaging of calcium fluorescence transients from individual dendritic spines in brain tissue in situ (Figure 3). Ca^2+^ transients evoked in spines via activation of Ca^2+^-permeable AMPA receptors are small and difficult to resolve in intact tissue. To test whether this was feasible using LLSM, CA1 pyramidal neurons were labeled with the fluorescent dye, Alexa 647, and the Ca^2+^-sensitive dye, Oregon Green BAPTA1, via a whole cell patch pipette. The neurons were first imaged in 3D and excited at 640 nm to identify dendritic structures (Figure 3a). To isolate signals to AMPA receptor function, the tissue was superfused with D-AP-5 (50 µM) to block NMDA receptors, and the cells were also filled with QX314 via the patch pipette to prevent dendritic spiking. During subsequent imaging at 488 nm excitation, a short train of stimuli (10 shocks at 100 Hz) was applied to the Schaffer collateral commissural pathway. Discrete sites of Ca^2+^ entry were observed (Figure 3b,c) coincident with dendritic spine-like structures consistent with the synaptic activation of receptors.

### 3.2. LLS Imaging of Single AP-Evoked Fluorescent Ca^2+^ Transients In Situ

**Figure 4:**
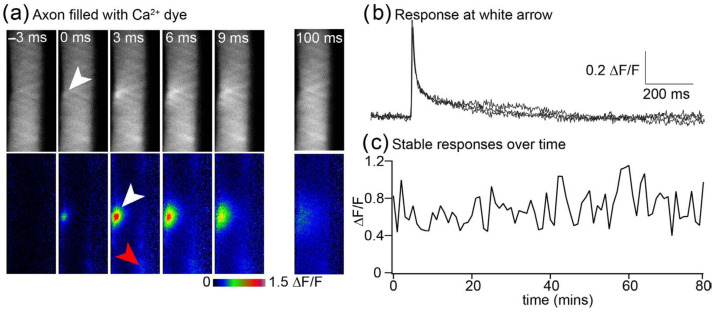
High-spatiotemporal-resolution imaging of Ca^2+^ transients at presynaptic terminals. (**a**) Lamprey giant axon in the intact spinal cord was recorded with a microelectrode to fill the axon with the Ca^2+^-sensitive dye Oregon Green BAPTA1. The axon was imaged at 3 ms intervals in a single LLSM plane and single action potential stimulated at 0 ms. In this example, the site of Ca^2+^ entry was in the plane of the LLSM (white arrows), although others were just out of focus (e.g., red arrow). The raw data with no dithering are shown (top, grey), and data are expressed as ΔF/F (in LUT colors, second row) for frames before and during the response. (**b**) Three superposed example traces showing the extraordinary resolution of these responses that are caused by between 1 and 6 voltage-dependent Ca^2+^ channels [6]. (**c**) The LLSM causes very little photodamage. Shown are the amplitudes of 80 sequential responses captured at 1 min intervals. Prior experiments using epifluorescence or confocal imaging of these responses cause substantial loss of signal due to photo-damage after 20 recorded responses [8].

Action potentials in lamprey reticulospinal axons causes highly localized Ca^2+^ entry to only occur at active zones [20], and we have demonstrated that few channels open at each AZ on presynaptic depolarization [6]. Prior methods that are sufficiently rapid to image Ca^2+^ transients generated by single action potentials, such as confocal line scanning [23], suffer from relatively high noise and photodamage. The LLSM technique demonstrated here [1] enabled optical slicing rapidly enough to resolve presynaptic Ca^2+^ transients (Figure 4). The much-reduced photobleaching [1] allowed the recording of many repetitions of stimulated transients. This has allowed us to investigate the stochastic nature of channel-dependent presynaptic Ca^2+^ entry [6] and will, in the future, enable the recording of stable responses to investigate synaptic plasticity. Here, we demonstrate the stability of recording sequential action potential-evoked transients.

We used LLSM to demonstrate the stability and resolution of Ca^2+^ signals at these terminals, in which we have shown that only between one and six Ca^2+^ channels contribute to the evoked response [6]. AP-evoked Ca^2+^ transients at AZs in situ in these axons were imaged at 330 Hz at a fixed z-axis (Figure 4a,b). Ca^2+^ transients occur as individually resolved hotspots (Figure 4a) that prior studies have demonstrated colocalize to AZs [20], although LLSM gave substantially greater signal-to-noise ratios (mean LLSM rms peak signal-to-noise ratios = 41.1 ± 14.2 n = 10, compared with 4.0 ± 1.3 with line scanning n = 6). LLSM reveals readily resolved transients at single AZs during each AP. To illustrate the response to single presynaptic AP, the response to one stimulus at a single hotspot is shown, as well as the time dependency of the transient intensity over time (Figure 4b). The graph represents the amplitude of sequentially recorded responses, showing that the mean amplitude is stable while individual amplitudes vary stochastically (Figure 4c).

### 3.3. Staining and Destaining of Synaptic Vesicle Clusters with FM1-43

**Figure 5:**
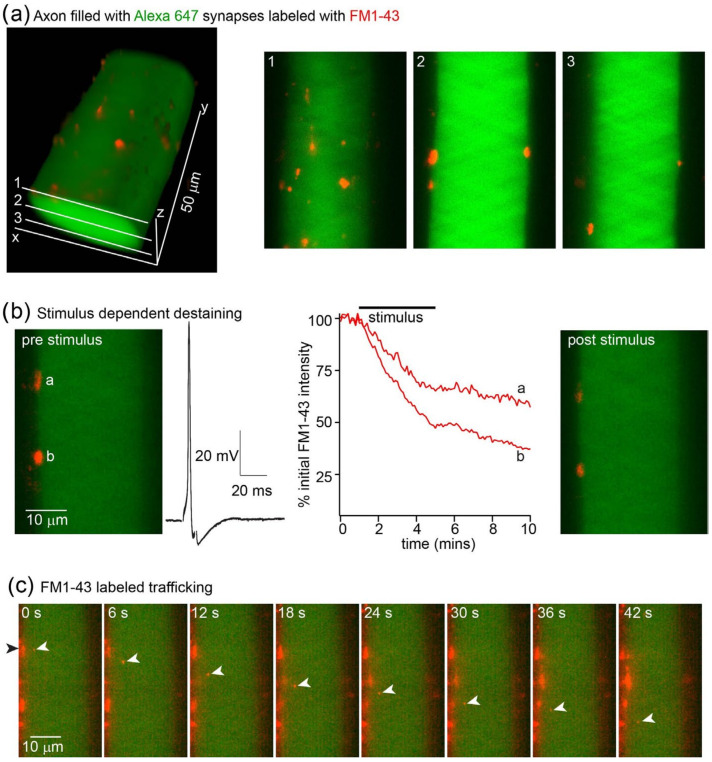
Vesicle staining and stimulus-evoked destaining in giant axons. (**a**) Lamprey giant axon in the intact spinal cord was recorded with a microelectrode containing Alexa fluor 647 hydrazide (green, excited with 640 nm excitation) visualized under LLSM. The axon was filled by pressure injection. During superfusion of the tissue with the dye FM1-43 (red), the axon was stimulated with 2000 action potentials via a depolarizing pulse applied through the microelectrode. After stimulation, excess FM1-43 was cleared from the tissue with Advasep-7 [21,22]. These revealed puncta imaged at 488 nm excitation around the surface of the axon shown in 3D (left) and for 3 planes (right). (**b**) The axon was stimulated (5 Hz, 2000 action potentials, 10 overlaid example traces shown, black), and the 50 planes at 1 mm z plane steps were captured continuously at 20 ms intervals. Shown on the left is a single plane before stimulation, highlighting 2 puncta. Graph (red) shows destaining during stimuli for these two puncta, labeled a and b. The right-hand image shows this plane after stimuli. (**c**) This panel emphasizes the spatio-temporal resolution of this imaging approach. Prior use of confocal microscopy demonstrated destaining, but during operation, LLSM reveals numerous components of puncta exiting the structures and being transported both anterogradely (example shown with white arrows) and retrogradely in the axon. Prior approaches had neither sufficient temporal resolution to track objects in 3D nor the sensitivity to resolve these objects.

Synaptic vesicle clusters can be stained in vitro in culture with the membrane dye FM1-43. This allows the subsequent tracking of rates of exocytosis by measuring the stimulus-dependent rate of the dye destaining from presynaptic terminals [8]. This approach has been extended to the staining of vesicle clusters in intact tissue, provided that excess dye is removed or quenched. One approach that has been successfully used is to use the cyclodextrin Advasep-7 as a chelating agent to extract excess dye from tissue [22]. Subsequently, the intensity of staining of the puncta comprising many synaptic vesicles can be measured using confocal microscopy [21]. Here, we demonstrate that these synaptic vesicle clusters can be imaged using LLSM. We used sharp microelectrodes to record from individual lamprey reticulospinal giant axons within the intact spinal cord during LLSM imaging (Figure 5). Axons were labeled with a long wavelength dye (Alexa 647 hydrazide) by pressure ejection from the microelectrode. In these axons, synaptic vesicles were labeled with the membrane dye FM1-43. Dye was superfused over the tissue and allowed to diffuse around the axons. Stimuli were applied through the recording microelectrodes to evoke 2000 action potentials at 5 Hz. Dye was removed from the superfusate, and excess dye was cleared from the tissue using Advasep-7 as a chelator [22]. This left labeled synaptic vesicles [24] (Figure 5a). LLSM enables stimulus-evoked exocytosis to be monitored by investigating rates of evoked destaining (Figure 5b), similar to earlier work [21], but in addition, the very high sensitivity of the approach made it possible to image the movement of lipids transported between vesicle clusters (Figure 5c).

### 3.4. IHLLS Quantitative 3D Neuronal Cell Imaging

The lamprey spinal cord was isolated and placed ventral side up to expose the reticulospinal axons closest to the imaging system in a cooled, small-volume chamber with a sylgard floor. The chamber and spinal cord were then transferred onto the customized stage of the LLS microscope. The recording chamber was continually superfused with cold, oxygenated Ringer (8–10 °C) for the duration of the experiment while maintained under the dual lenses of the LLS system. Axons were labeled similarly to those shown in Figure 4, except that for these experiments, axons were recorded with sharp electrodes that contained the lipophilic dye FM1-43 (20 μM). Injection of the dye directly labeled membranes in the intracellular spaces, including endoplasmic reticulum, mitochondria, endosomes, and synaptic vesicle clusters. Visible labeling was limited to where the dye interacted with the membrane because FM1-43 is not fluorescent in aqueous solution but becomes fluorescent when interacting with lipids. This gave a stable structure to highlight the capabilities of IHLLS imaging.

**Figure 6:**
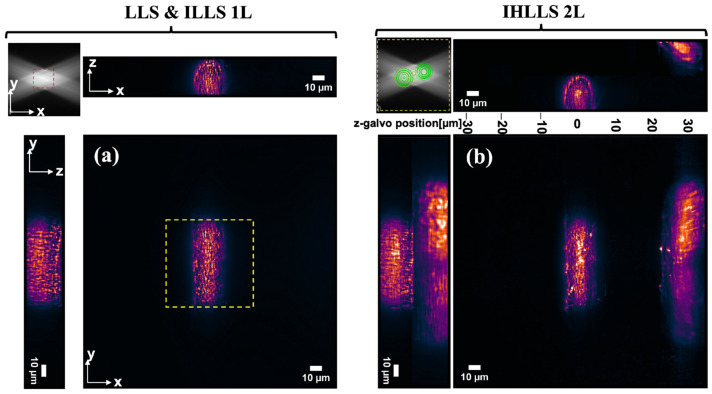
(**a**) Tomographic imaging of a lamprey spinal cord ventral horn neuron with axons (*xy, FOV* 208 × 208 µm^2^, 2048 × 2048 pixels; *yz, (xz) FOV* 208 × 30 µm^2^, 2048 × 300 pixels) in a conventional LLS and IHLLS 1L without deconvolution. On the sides and above are shown the max projections through the volume (300 z-galvo steps). The Bessel beams are displayed in the upper-left corner of the xy-projection to show the orientation of the beams (*FOV* 208 µm^2^). The area enclosed inside the red dashed rectangles is the scanning area for the original LLS (52 µm^2^). (**b**) Tomographic imaging of a lamprey spinal cord ventral horn neuron with axons (*xy, FOV* 208 × 208 µm^2^, 2048 × 2048 pixels; *yz, (xz) FOV* 208 × 60 µm^2^, 2048 × 600 pixels) in IHLLS 2L without deconvolution. On the sides and above are shown the max projections through the volume (in this case, 5 z-galvo steps). The Bessel beams are displayed in the upper-left corner of the xy-projection to show the orientation of the beams, including some diffraction patterns caused by the phased SLM. The area enclosed inside the yellow dashed rectangles is the scanning area for the IHLLS 2L (208 µm^2^).

We carried out the collection of tomographic LLS and IHLLS 1L data using the conventional LLS pathway or the IHLLS 1L, respectively, where the *z-galvo* and *z-piezo* were stepped in $\delta z_{\mathrm{LLS}}=0.101\;\mu m$ increments through the focal plane of the emission objective, for a displacement range of $\Delta z_{\mathrm{galvo}}=30\;\mu m$ (Figure 6a) for a scanning area of $208\times 208\;{\mu m}^{2}$. The IHLLS 1L set of images was obtained by projecting a diffractive lens of focal length $f_{\mathrm{SLM}}=415\;\mathrm{mm}$ on the phase SLM. Since the IHLLS 1L and LLS have the same scanning geometry and almost identical imaging performances, we displayed only one set of images in Figure 6a. As we can notice, both systems, using the same diffraction anulus of NA_out_ = 0.55 and NA_in_ = 0.48 in the excitation path of the LLS, cannot scan outside the red dashed rectangle area, which is 52 µm^2^. Therefore, this anulus filter cannot be used to image larger objects such as axons without using auxiliary scanning methods, e.g., tiling [25]. The scanning area is linked to the light sheet length, which is an overall combination of the two NAs of the annular mask filter, NA_out_ and NA_in_ [26]; nevertheless, any of these combinations will not cover the FOV of the detector. The Bessel-like beams are considered to be created using those annuli on the LLS diffraction mask with NA_out_ between 0.5 and 0.64, and the Gaussian-like beams are created with the annuli with NA_out_ between 0.2 and 0.4. It is hard to compress the light sheet thickness using Gaussian-like beams while maintaining a relatively large FOV, and it is also hard to align a system with NA_out_ of 0.64 using Bessel-like beams with longer light sheets. The approach of using holographic techniques with optical sectioning, achieved by moving the light sheet at various depths inside the sample, provides both functional and structural information about intracellular events, cells, cell–cell interactions, tissues, and larger organisms. Using the IHLLS 2L (Figure 6b), the scanning area is expanded to the full FOV of the detector. Another improvement of using the IHLLS 2L is that the volume of the sample is scanned with fewer z-galvo steps. In this case, the *z-galvo* was moved in increments of 10 μm between −10 μm and 30 μm. In this case, the electrode was pointing from the left toward the center of the FOV, and the sample did not have any axons filled with dye below the *z-galvo =* −10 μm position; therefore, we performed the scanning above the −10 μm position. The spanning volume between each two *z-galvo* levels is recovered numerically by using a phase shifting technique (θ=0, θ=π/2, θ=π,θ=3π/2), together with a diffraction algorithm. The results are summarized in Figure 6b, which depicts the xy map projections and cross-sections of the sample volume. We noticed only two axons were imaged in the maximum FOV of the detector, one localized around the $z_{\mathrm{galvo}}=0\;\mu m$ and the second one localized between 20 μm and 30 μm.

## 4. Discussion and Conclusions

LLSM microscopy represents a very sensitive, low photodamage, and high-speed system for imaging at subcellular resolution [1]. Many of the attributes of the system are equally applicable for imaging in situ. Compared to light sheet imaging with Gaussian beams, the LLSM approach shows relatively good tissue penetration. Satisfactory images that, for example, enabled synaptic spine images were feasible at depths in tissue up to 50 µm (Figure 4; see also reference [7]). Thus, although the approach does not have the depth recording capabilities of multiphoton imaging, it provides distinct advantages for imaging in tissues. These include the speed of volumetric imaging while retaining high sensitivity. Thus, for example, following labeling of endocytosed vesicles with FM1-43 it is possible to track transported membrane structures originating from the synaptic vesicle clusters. Other examples are approaches that provide very high temporal imaging at fixed focal planes. We demonstrated two such recording paradigms in which Ca^2+^ transients are captured at high frame rates in pre- or post-synaptic structures. These approaches overcome many of the limitations of high-speed confocal or multiphoton line scan imaging—an approach with limited spatial information and very high rates of bleaching and photodamage.

For many of these approaches, it is also advantageous to combine imaging with electrophysiological techniques. This enables the correlation of electrical signals and stimuli to be made directly with images of dynamic changes in the cells. This is relatively straightforward with LLSM, because electrodes can be introduced in the gap between the excitation and emission objectives. Electrical interference from dipping lens contact with the superfusate was eliminated by coating the lens tubes with wax, and the standard LLSM chamber was replaced with an open, continuously perfused chamber design. Focusing the emission plane for this purpose was achieved via piezo control of the lens position, because moving the sample would prevent electrical stimulation and recording.

It is advantageous not to move the emission lens. To enable this, we have also demonstrated that our incoherent digital holographic system could be used in the imaging of intact tissue with a resolution comparable or better than the conventional LLS system. Moreover, the system can achieve a full FOV and deeper scanning depth by modulating the wavefront of the emission beam with the diffractive lenses uploaded on the phase SLM. We have shown that this approach is capable of reconstructing structures that are involved in dynamic changes measured with the LLS approach. We are currently developing the ability to measure tomographic time series images with holography to rapidly track dynamic changes in 3D space.

## Data Availability

Supporting data are available from the corresponding author on reasonable request. This is due to the size of the datasets being so large that they are not available on a public server.
